# Mild cold effects on hunger, food intake, satiety and skin temperature in humans

**DOI:** 10.1530/EC-16-0004

**Published:** 2016-03-01

**Authors:** M Langeveld, C Y Tan, M R Soeters, S Virtue, G K Ambler, L P E Watson, P R Murgatroyd, V K Chatterjee, A Vidal-Puig

**Affiliations:** 1 University of Cambridge Metabolic Research LaboratoriesWellcome Trust-MRC, Institute of Metabolic Science, Addenbrookes Hospital, Cambridge, UK; 2 Cambridge Vascular UnitAddenbrookes Hospital, Hills Road, Cambridge, UK; 3 NIHR/Wellcome Trust Clinical Research FacilityAddenbrookes Hospital, Cambridge, UK

**Keywords:** Cold, thermogenesis, hunger

## Abstract

**Background:**

Mild cold exposure increases energy expenditure and can influence energy balance, but at the same time it does not increase appetite and energy intake.

**Objective:**

To quantify dermal insulative cold response, we assessed thermal comfort and skin temperatures changes by infrared thermography.

**Methods:**

We exposed healthy volunteers to either a single episode of environmental mild cold or thermoneutrality. We measured hunger sensation and actual free food intake. After a thermoneutral overnight stay, five males and five females were exposed to either 18°C (mild cold) or 24°C (thermoneutrality) for 2.5 h. Metabolic rate, vital signs, skin temperature, blood biochemistry, cold and hunger scores were measured at baseline and for every 30 min during the temperature intervention. This was followed by an ad libitum meal to obtain the actual *desired* energy intake after cold exposure.

**Results:**

We could replicate the cold-induced increase in REE. But no differences were detected in hunger, food intake, or satiety after mild cold exposure compared with thermoneutrality. After long-term cold exposure, high cold sensation scores were reported, which were negatively correlated with thermogenesis. Skin temperature in the sternal area was tightly correlated with the increase in energy expenditure.

**Conclusions:**

It is concluded that short-term mild cold exposure increases energy expenditure without changes in food intake. Mild cold exposure resulted in significant thermal discomfort, which was negatively correlated with the increase in energy expenditure. Moreover, there is a great between-subject variability in cold response. These data provide further insights on cold exposure as an anti-obesity measure.

## Introduction

At first sight obesity may appear as a condition that is easy to treat by either decreasing energy intake and/or increasing the energy expenditure. In practice, long-term weight loss is very difficult to achieve. Since strategies that reduce energy intake fail in the majority of patients, increasing energy expenditure seems to be an attractive alternative. Energy dissipating drugs (e.g., thyroid hormone, ephedrine, dinitrophenol) have been used successfully to decrease body weight, but their use was discontinued because of unacceptable cardiovascular side effects (1, 2, 3). Exercise may be a healthier approach to increase energy expenditure, but the amount of exercise needed to significantly influence energy balance, as well as the accompanying increase in appetite, makes it an ineffective strategy for long-term body weight reduction (4).

Exposure to cold increases energy expenditure and is partly mediated by the activation of brown adipose tissue (BAT). Non-shivering thermogenesis (NST) is the increase in energy expenditure resulting from exposure to temperatures below the thermoneutral zone, but above the temperature threshold for shivering. By definition, no physiological mechanisms for temperature regulation are active at thermoneutrality and therefore no energy is spent on temperature maintenance. For naked humans, the thermoneutral zone is 27 ± 2°C (5) and for lightly clothed humans it lies around 22–24°C, depending on the insulative properties of the clothing (6).

As described above, cold exposure induces physiological changes. More importantly, mild cold exposure may have a better adherence compared with profound cold exposure when used as an anti-obesity strategy to increase the metabolic rate. The key question is whether increasing energy expenditure through mild cold exposure is accompanied by an increase in appetite and food intake. Cold exposure is known to increase food and energy intake in a wide range of animal species, e.g., piglets, rats and birds (7, 8, 9), but not in humans.

Besides the increase in energy expenditure, cold exposure may also trigger an insulative response. Vasoconstriction, mediated via activation of alpha-adrenergic receptors, limits heat loss via the skin. Interestingly, the vasoconstrictive response is highly variable between individuals and was shown to correlate negatively with the magnitude of NST in one study (10). Skin temperature changes during cold exposure may thus be a predictor of the metabolic response to cold exposure. This may also be the case for changes in the temperature of the skin overlying the supraclavicular BAT depot, as suggested by two reports using infrared thermography to measure skin temperature in the supraclavicular region during a cold challenge (11, 12). Moreover, it is the reduction in skin temperature during cold that is mediated by vasoconstriction, which is perceived as uncomfortable.

In this study, we investigated the response to mild cold in healthy humans for this may be an attractive weight management strategy. More importantly, we focussed on changes in energy expenditure, food intake including appetite and satiety, and dermal temperature.

## Subjects and methods

### Subjects

Healthy volunteers were recruited through local advertisements in the East Anglian region of the United Kingdom. We recruited five lean males and five lean females, non-smokers, aged between 22 and 60 years, who had no known medical conditions and were not taking any medications or supplements. To minimise seasonal variation of NST, which is known to exist, subjects were studied between April 2012 and September 2012 (13). All subjects provided written informed consent and the study conformed to the standards set by the latest revision of the Declaration of Helsinki. The study received approval from the Cambridge Central East of England Research Ethics Committee.

### Study outline

**Figure 1:**
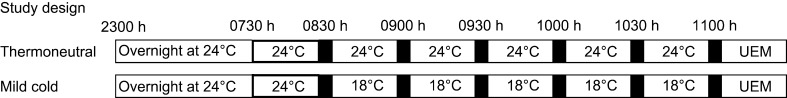
Study design. Black bars represent measurements including indirect calorimetry, cold and hunger scores, vital signs and blood test. UEM, universal eating monitor.

The outline of the study design is depicted in Fig. 1. The subjects were studied twice during two days, about two weeks apart, one of the days the subjects were tested under thermoneutrality and the other day under mild cold exposure, they were blinded to the setting and tests were performed in a random order. The subjects were asked to refrain from strenuous physical activity, alcohol and caffeine for 24 h before their visit. Each participant arrived at the Clinical Research Facility around 16:00 h on day 0 and remained until 14:00 h on day 1. Height, weight and body composition (DXA (GE Lunar Prodigy GE Healthcare, Madison, WI, USA; software version 12.2)) were measured. At 18:00 h, a standardised dinner was served. The energy content of the meal was 1/3 of a participant's daily requirements estimated from predicted resting metabolic rate, using the Schofield equation, multiplied by an activity factor of 1.35. Meal composition was 30–35% fat, 12–15% protein and 50–55% carbohydrate by energy. The participants retired to bed in the temperature controlled room (24°C) at 23:00 h and were provided with standardised light clothing and bedding. The temperature controlled room is a habitual room including a desk, television, computer, sink and toilet. The participant was woken the next morning at 07:00 h and stayed in bed in a semi-supine position (upper part of the bed at 45°C) without bedding. All participants were asked to remain awake and inactive. To enable thermal imaging, male subjects had a bare torso and women wore a boob tube for the remainder of the experiment. Baseline indirect calorimetry, vital signs, cold and hunger scores and blood tests were taken at 7:30 h. Next, the subjects either stayed in this room at 24°C or were moved to the mild cold room (18°C). Subsequently, thermal imaging, vital signs, indirect calorimetry, cold and hunger scores and blood tests were repeated every 30 min (Fig. 1) during 2.5 h. In between the measurements, the subjects were allowed to read or watch TV but did not leave the bed except for toileting. Blood samples were drawn via a large indwelling venous catheter without using a heated hand box or blanket to prevent local warming. Afterwards, the universal eating monitor was used to assess the speed of eating ad libitum meal and other parameters related to appetite and food intake.

### Indirect calorimetry

REE was measured by ventilated canopy respiratory gas exchange (GEM; GEMNutrition, Daresbury, UK) in a supine position. The measurements were recorded during 12-min intervals for every 30 min. Energy expenditure was calculated from the macronutrient respiratory quotients and energy equivalents of oxygen published by Elia and Livesey (14).

### Cold and hunger scores

The participants were asked to rate the sensation of cold of the whole body and hands separately on a 1–10 scale, with ratings as follows: 1 was rated as ‘not at all cold’ and 10 was the ‘coldest one had ever felt’. Similarly for the degree of hunger, with ratings as: 1 for ‘not hungry at all’, and 10 was rated as ‘the most hungry one had ever felt’.

### Blood biochemistry

Glucose was measured by Hexokinase method on a Siemens Dimension RXL AutoAnalyser. Reagents and Calibrators were purchased from Siemens. Free fatty acids were measured using Roche Free Fatty Acid Kit. This assay was modified to run in a microtitre plate format. Thyroid-stimulating hormone (TSH), free thyroxin (fT4) and iodothyronin (T3) were measured by time-resolved fluorescence immunoassay on an AutoDELFIA analyser (Perkin Elmer) using kits from Perkin Elmer. Cortisol level was measured by fluorescence immunoassay on the Siemens Centaur Autoanalyser. A minimum of two quality control samples were run in each assay.

### Universal Eating Monitors (UEMs)

The UEM (The Sussex Meal Patterning System) was used. The subjects ate an homogenous test meal (e.g., pasta) containing normal energy percent ratios (∼30% carbohydrates, ∼30% protein and ∼40% fat). Intake of test meal was continuously monitored using the UEM equipment (15). Herein, food is served and eaten from a plate placed on weighing scales connected to a computer. The generated intake data contained the amount eaten and the seconds spent on eating. The monitors allow automated combinations of appetite ratings by the visual analogue scales and intake data. The VAS scales rate feelings of hunger, sickness, fullness and desire to eat on a 0–100 scale (16).

### Thermography

Skin temperature images were obtained using a ThermaCam 3000, and images were analysed by ThermaCAM Researcher Pro 2.9 software (both FLIR systems, Boston, United States). The camera settings were temperature dependent: at 24°C; emissivity: 0.98, humidity: 45%, distance: 1.2 m, external and reflected temperature: 24°C, at 18°C, emissivity: 0.98, humidity: 40%, distance: 1.2 m, and external and reflected temperature: 18°C. Two skin regions were defined: first the supraclavicular area (bordered by the acromioclavicular joint, sternoclavicular joint, and the sternocleidomastoid trapezoid angle) and secondly, the sternal area (the top 10 cm above of the sternum). For recognition of these anatomical landmarks on the thermal images, we placed metal markers on the skin. At each time point, per area the average of three images was taken for analysis.

### Statistical analyses

All analyses were performed using SPSS 21. Time series data were analysed using repeated measures ANOVA. Each ANOVA model was built using ‘time’ as within-subject effect, ‘temperature’ as independent factor, and ‘time*temperature’ as the interacting term. A significant ‘time*temperature’ effect is interpreted as a significant effect of mild cold exposure on the rate of change over time. Each term in the ANOVA model was analysed for sphericity (Mauchly’s Test) and if found to be violated, within-subject effects was determined by the Greenhouse-Geisser test. For all statistical test, a *P* value of <0.05 was considered to be significant. All paired data were analysed by Student's *t*-test. The correlations were assessed using Pearson's test. Data are presented as mean ± S.D.

## Results

### Metabolic response to mild cold exposure

**Figure 2:**
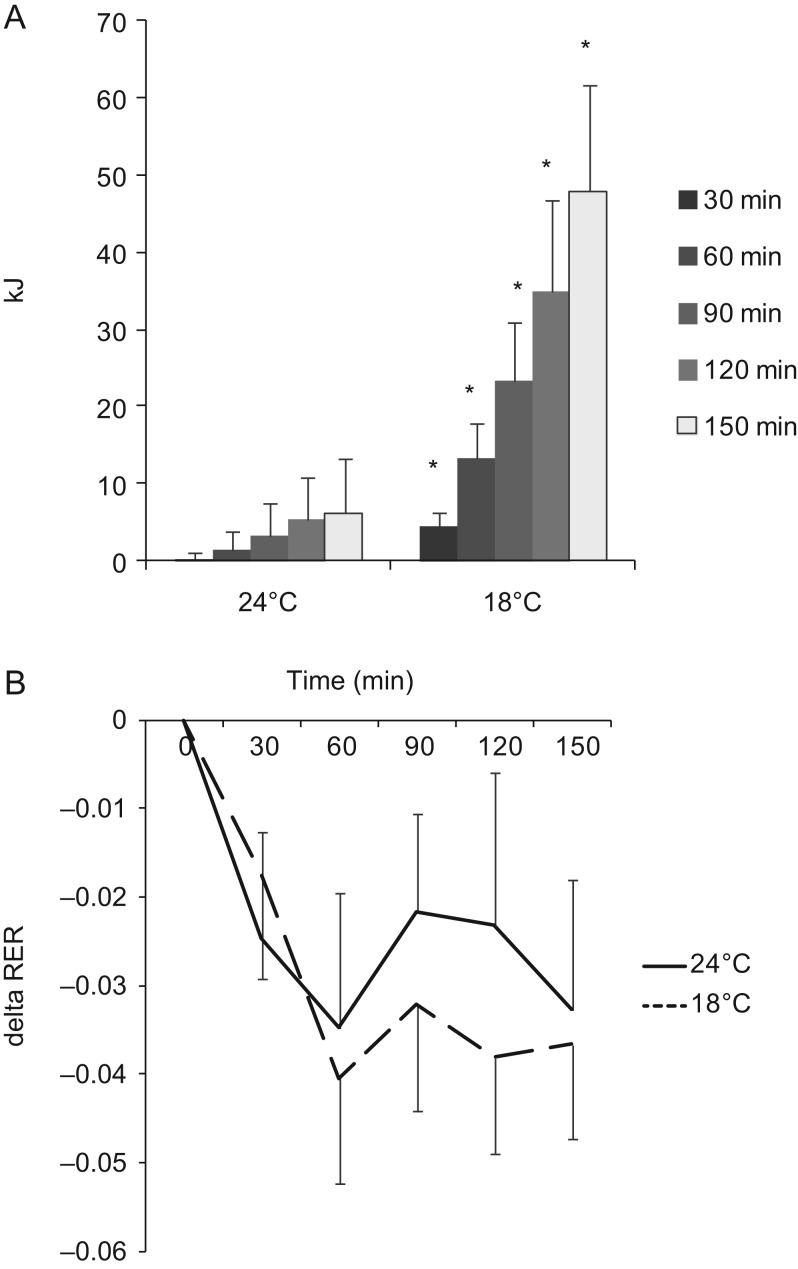
(A) Cumulative energy expended above basal metabolic rate over 150 min of thermal challenge. *P < 0.05 compared with 24°C. (B) Change in respiratory exchange ratio (RER) compared with baseline.

We studied 10 Caucasian healthy subjects, 5 males and 5 females, age ranging from 22 to 60 years old, BMI ranging from 20.8 to 24.9 kg/m^2^. The characteristics of the subjects are included in Table 1. Mild cold exposure significantly increased energy expenditure without visible shivering compared with thermoneutrality (repeated measurement ANOVA for cold effect *P* = 0.01). Over 150 min of exposure to 18°C, a total of 48 ± 14 kJ (range 13–127 kJ) was expended above the baseline energy expenditure at 24°C (Fig. 2A). Respiratory exchange ratio (RER) dropped during the experiment under both conditions (repeated measurement ANOVA for the effect of time *P* < 0.01). There was no significant effect of temperature on RER (Fig. 2B; repeated measurement ANOVA for the effect of temperature *P* = 0.195).

**Table 1:** Characteristics of subject

	**Males**	**Female**
Age (years)	44.7 ± 5.2	33.7 ± 6.9
BMR (J/min)	4445.5 ± 225.9	3808 ± 196.6
Height (m)	1.76 ± 0.03	1.65 ± 0.04
Weight (kg)	69.5 ± 3.3	62.6 ± 4.1
BMI (kg/m^2^)	22.4 ± 0.8	22.9 ± 0.9
Fat (kg)	12.8 ± 2.7	20.2 ± 2.4
Lean (kg)	53.1 ± 1.8	39 ± 2.2
FFM (kg)	55.8 ± 1.9	41.5 ± 2.4

BMR, basal metabolic rate; BMI, body mass index; FFM, fat free mass.

### Vital signs

**Figure 3:**
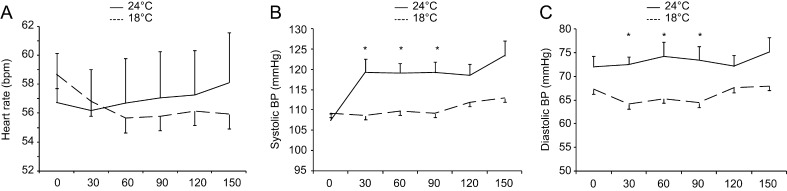
(A) Average heart rate, (B) systolic blood pressure and (C) diastolic blood pressure over 150 min of thermal challenge. *P < 0.05 for 24°C vs 18°C.

Heart rate remained stable at thermoneutrality at 56.7 ± 4.2 (*T* = 0) to 58.1 ± 4.2 (*T* = 150 min) beats per minute (bpm). Heart rate decreased in response to mild cold exposure from an average of 59.5 ± 4.4 (*t* = 0) to 56.7 ± 4.5 bpm (*t* = 150 min) (Supplementary Figure 1A, see section on supplementary data given at the end of this article; repeated measurement ANOVA for the effect of time*temperature *P* = 0.025). Systolic blood pressure remained stable between 109 ± 2 (*T* = 0) and 113 ± 2 mmHg (*T* = 150 min) at thermoneutrality and increased from 107 ± 2 (*T* = 0) to 120 ± 4 mmHg (T = 150 min) during mild cold exposure (Fig. 3; repeated measures ANOVA for the effect of time*temperature *P* = 0.007). Diastolic blood pressure remained stable during both the stay at thermoneutrality and at mild cold exposure (Fig. 3).

### Biochemistry

**Figure 4:**
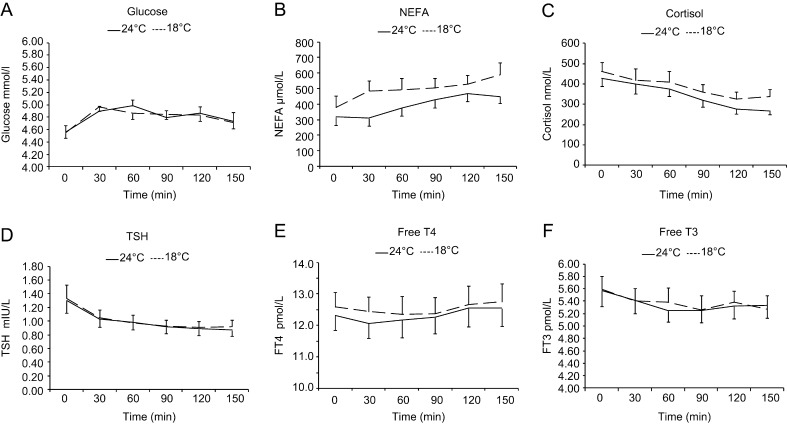
(A) Plasma glucose, (B) non-esterified free fatty acid (NEFA), (C) cortisol, (D) thyroid-stimulating hormone (TSH), (4) free thyroxine (FT4) (E) and free tri-iodothyronine (FT3) (F) over 150 min of exposure to 24°C and 18°C.

Plasma concentrations of glucose and fT4 increased similarly, and plasma cortisol, TSH and T3 concentrations decreased similarly under both thermal conditions (Fig. 4). The only biochemical parameter that responded to a difference in ambient temperature was the plasma non-esterified fatty acid (NEFA) levels (Fig. 4B). NEFA levels increased under both conditions, but this increase was more at mild cold exposure (211 ± 62 μmol/L) compared with thermoneutrality (132 ± 59 μmol/L) (repeated measures ANOVA for the effect of temperature *P* < 0.001).

### Cold sensation

**Figure 5:**
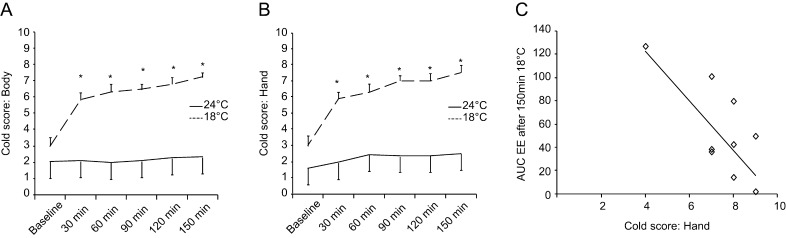
(A) Score for the perception of cold (cold score) for whole body and (B) hands. (C) Correlation between cold score for hands and EE over baseline after 150 min of mild cold exposure.

At thermoneutrality, the score for whole body cold sensation remained stable between 2.1 ± 0.5 and 2.3 ± 0.5, whereas during mild cold exposure the score significantly increased from 3.0 ± 0.5 to 7.2 ± 0.3 (repeated measures ANOVA for the effect of temperature *P* < 0.001) (Fig. 5A). The same pattern was observed also for hand, at thermoneutrality no significant change, but during mild cold exposure the cold score increased significantly from 3.0 ± 0.6 to 7.5 ± 0.5 (Fig. 5B, repeated measures ANOVA for the effect of temperature *P* < 0.001). There was a significant negative correlation between the cold score for hands after 150 min of mild cold exposure and the cumulative increase in energy expenditure above baseline during this time (Fig. 5C, *r*^2^ = 0.481, Pearson *P* < 0.001). There was no correlation between the cold score for whole body at 150 min and the cumulative increase in energy expenditure in response to mild cold (*r*^2^ = 0.000).

### Hunger, food intake and satiety

**Figure 6:**
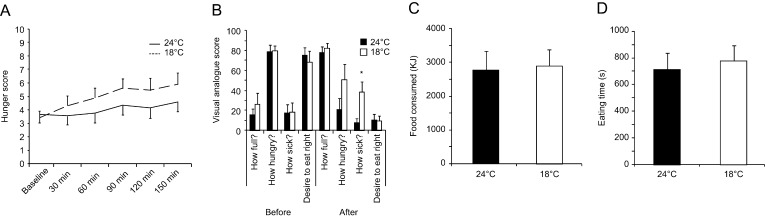
Score for perception of hunger over 150 min of thermal challenge. (A) Visual analogue scale for hunger and satiety before and after UEM test meal (B) Amount of food consumed (KJ) (C) and time spent eating (seconds) (D) during UEM test meal. *P < 0.05 for 24°C vs 18°C.

Feelings of hunger increased during both situations over 150 min. A trend towards a higher hunger score during cold was observed (Fig. 6A, repeated measurement ANOVA for the effect of time *P* = 0.021, for the effect of temperature *P* = 0.064). Before the meal, feelings of fullness, hunger, sickness, and desire to eat were similar after exposure to both thermal conditions (Fig. 6B). During the meal, the same amount of food was consumed after thermoneutrality and mild cold exposure (2740 ± 567 vs 2878 ± 492 kJ paired *t*-test *P* = 0.69) (Fig. 6C). There were no differences in the time spent eating during both situations: thermoneutrality (714 ± 124 s) versus cold (778 ± 115 s) (paired *t*-test *P* = 0.14) (Fig. 6D). There was no correlation between the amount of food consumed and basal metabolic rate, and between the amount of food consumed and the increase in energy spent during 150 min of mild cold exposure (*r*^2^ = 0.076, *P* = 0.271 and *r*^2^ = 0.00, *P* = 0.962 Pearson test). Feelings of fullness, hunger, and desire to eat after the test meal were not different after either situation: cold versus thermoneutrality (Fig. 6B). Feeling of sickness was significantly greater after mild cold exposure (Fig. 6B).

### Skin temperature changes assessed by thermography

**Figure 7:**
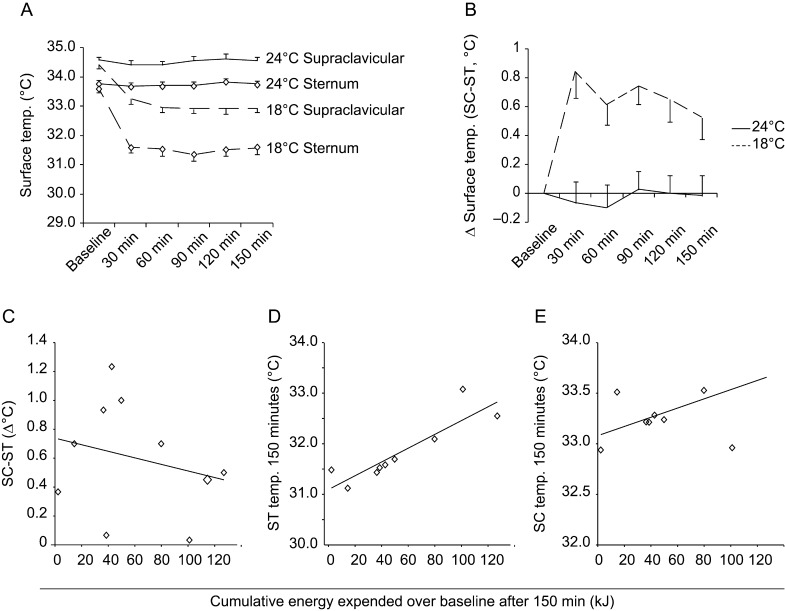
Surface temperature over the supraclavicular and sternal area as measured by thermal imaging at 24°C and 18°C (A) over 150 min of thermal challenge. (B) Temperature difference between supraclavicular area and sternal area. (C) Correlation between cumulative energy expended (EE) over baseline after 150 min of mild cold exposure and the change in temperature difference between supraclavicular and sternal areas after 150 min. (D) Correlation between EE over baseline after 150 min of mild cold exposure and sternal temperature after 150 min. (E) Correlation between EE over baseline after 150 min of mild cold exposure and supraclavicular temperature after 150 min.

During both situations (thermoneutrality and cold exposure), the supraclavicular skin temperature was higher compared with the sternal skin area (repeated measurement for the effect of location ANOVA *P* < 0.01). At 24°C skin temperature in both areas remained stable (Fig. 7A). In response to mild cold exposure, skin temperature dropped in both areas during the first 30 min and stayed stable thereafter. This temperature drop was greater in the sternal area compared with the supraclavicular area (Fig. 7A). The temperature difference between both areas increased in response to mild cold exposure and remained stable at thermoneutrality (Fig. 7B; repeated measurement ANOVA for the effect of temperature*time interaction *P* < 0.01). There was a tight positive correlation between the skin temperature of the sternal area and the increase in energy expenditure (Fig. 7D; *r*^2^ = 0.787, *P* = 0.001, Pearson test). Supraclavicular temperature was not correlated with the increase in energy expenditure (Fig. 7E; *r*^2^ = 0.286, *P* = 0.103, Pearson test).

## Discussion

In this paper, we investigated the response to mild cold in healthy humans with a focus on changes in energy expenditure, food intake, and dermal temperature. As described in previous studies, exposure to mild cold induced a small but significant increase in energy expenditure. We showed that, at least in short-term, energy intake does not increase since two and a half hours of mild cold exposure had no influence on the amount of food eaten and time spent eating. During cold exposure, feelings of hunger showed a trend to increase to a level above what was observed during thermoneutrality. Once out of the cold, the difference disappeared and no differences could be found in pre-meal feelings of hunger, desire to eat, fullness or sickness after exposure to the two different thermal conditions. We cannot exclude the possibility that excess energy expenditure in cold is compensated by increased food-intake later. Whether prolonged mild cold exposure, with meals consumed in the cold, would increase energy intake remains to be determined. Historical data obtained under harsher thermal conditions show a negative correlation between outdoor temperature (ranging from −30 to +35°C) and food intake (9). To our knowledge, there are no data on the effect of prolonged mild cold exposure on hunger and food intake.

Skin temperature falls in response to a drop in ambient temperature. In the studies published so far, it was found that the supraclavicular temperature either increased (12, 17) or decreased to a lesser extend compared with other body areas (11, 18). A recently published study has shown a positive correlation between the skin temperature in the supraclavicular region and clavicular BAT volume and activity during cold exposure (17). Based on these data, we proposed a hypothesis that the change in the temperature difference between the supraclavicular region and the sternal area would be positively correlated with the increase in energy expenditure in response to mild cold exposure. This was not the case, nor was the supraclavicular temperature in itself related to the metabolic response to mild cold. We conclude that measurements of skin temperature in the areas overlying the superficial BAT depots are not helpful in predicting the metabolic responses to cold.

Relative mild cold exposure (18°C) resulted in significant thermal discomfort but no visible shivering in our study (Fig. 4A and B). This thermal discomfort is also likely to be perceived in daily life, since the average living room temperature in the UK has increased from approximately 18 to 21°C over the last three decades (18). Mild cold exposure is uncomfortable due to the perceived reduction in skin temperature, which is mediated by vasoconstriction. We not only show that a negative correlation between the metabolic and the vasoconstrictive response to mild cold exists, but also that the metabolic response to mild cold is negatively correlated with the cold score for hands. Therefore, the lower the metabolic response to mild cold exposure, the colder one feels. At extreme ends, individuals could be classified as ‘vasoconstrictors’; those with a strong insulative response and a low metabolic response to mild cold exposure and ‘metabolisers’; those with a high metabolic response that allows a relatively high rates of peripheral heat loss by maintaining a higher skin temperature. Hypothetically, vasoconstrictors would be at greater risk to develop overweight or obesity, since they will take behavioural measures to avoid the negative sensation elicited by mild exposure and spent less energy on thermoregulation compared with metabolisers.

As described earlier, mild cold exposure resulted in an increase in plasma free fatty acid (FFA) concentrations (e.g., 15, 19, 20). Studies on more extreme cold exposure have shown an increased rate of lipolysis in humans (21), making this the most likely source of increased FFAs during mild cold exposure. Mild cold exposure in humans increases systolic blood pressure (e.g., 15, 22, current study) and may increase LDL cholesterol concentrations (23, 24). Taken together, short-term mild cold exposure results in unfavourable changes in several cardiovascular risk factors. Lower temperatures are known to increase the incidence of cardiovascular events such as myocardial infarction and stroke, which have an increased incidence rate in winter, even in countries with milder climates (25). Whether the changes in cardiovascular risk factors that occur when lowering temperature in the range from around 22°C to around 16–18°C also results in a higher cardiovascular disease incidence remains to be established.

We aimed to standardise the experimental circumstances as much as possible. Therefore, subjects were admitted the afternoon before the study and exposed to exact identical meals, temperatures and sleep time. However, some limitations remain. Due to the intensive study protocol we only included 10 subjects, which may have prevented us from finding smaller effects. This may also explain why we did not find differences between men and women (data not shown) although the latter both may have more BAT and insulative capacity (26, 27, 28). Also the time spent in the cold and the temperature in which the meal was consumed may have led to results that are not generalisable. In addition, we did not measure BAT FDG uptake, but our primary aim was to investigate skin temperature in relationship with energy expenditure. Finally, we included healthy non-obese subjects and our results may not apply to obese subjects who may have larger insulative capacity due to more subcutaneous adipose tissue.

In conclusion, short-term mild cold exposure results in an increase in energy expenditure that is not directly compensated by an increase in energy intake and may thus be used to alter energy balance (29). The increase in energy expenditure is small, but if maintained throughout longer periods could be used to prevent weight gain or even promote modest weight loss. However, lower temperatures lead to thermal discomfort, especially in those with a low metabolic response to cold and may induce unfavourable cardiovascular and metabolic changes. Moreover, the response to cold is variable and not all subjects may show an increase in energy expenditure during cold exposure. Long-term studies measuring the effect of lowering ambient temperature on thermal comfort, body weight, adiposity and cardiovascular risk factors will have to establish whether mild cold exposure is an effective anti-obesity measure.

## Supplementary data

This is linked to the online version of the paper at http://dx.doi.org/10.1530/EC-16-0004.

## Declaration of interest

The authors declare that there is no conflict of interest that could be perceived as prejudicing the impartiality of the research reported.

## Funding

The study was funded by NIHR, BRC Seed Fund, individual grants: ML and MS: Marie Curie Fellowship, CYT: Welcome Trust Fellowship, SV: MRC, BHF and BBSRC, AVP: BBSRC.
